# Cochrane Colloquium in São Paulo

**DOI:** 10.1590/S1516-31802006000600001

**Published:** 2006-11-01

Humanity incorporates obvious ideas with difficulty and therefore slowly. But there is no doubt that this will be the century of technological evaluations in the field of health. How can tests and products for which effectiveness, efficiency and safety have not been demonstrated be incorporated into our diagnostic and therapeutic procedures? As you well know, dear reader, this is still done openly, which cannot be anything other than irresponsibility. In this equation, life does not necessarily come in first place! In fact, it is high time for this easily understood matter to be taken on board: life comes in first place.

But going back to technological evaluations, my question would be: who is it up to, to prove that a new car works (efficacy), works well in the places you go to (effectiveness), is economical and easy to maintain (efficiency) and is safe? Clearly, you are not going to pay to check on this: companies test their cars because they prefer to avoid greater problems with their consumers, and in particular with the law. Interestingly, the same thing does not take place in the field of health. Products are launched, sometimes at most on the basis of small clinical trials, and it is up to consumers to risk their health through medical prescriptions, to see whether the new item works or not.

However, this is the role of controlled clinical trials and the systematic reviews that sum them when they are similar and of good quality, in order to reduce the uncertainties in the questions of efficiency and safety. In the field of health, the consumer maxim "satisfaction guaranteed (cure) or your money (or life) back!" has no validity.

Recent history has demonstrated that the requirements of the United States Food and Drug Administration (FDA) have been insufficient to ensure that new products would cause more benefits than harm. Thus, it will be necessary for technological evaluations within the field of health to be developed and start to form part of the scientific activities in this field. Theoretical hypotheses in the era of the logic based on physiopathology need to be left behind for the era of logic that is proven or refuted by evidence. This will require an era of technological evaluations within the field of health that are based on reliable scientific evidence.

From October 23 to 28, 2007, the World Colloquium of the Cochrane Collaboration will take place in São Paulo. Researchers from more than 70 countries will discuss how to move towards "Evidence-based health for all". This theme was chosen to run alongside "Outcomes (results) based on patients’ preferences".

Who is it of interest to, that new technologies are evaluated on the basis of evidence? Firstly, people who have health problems (do you know anyone like this?). It is also of interest to people who wish to preserve their health. It is of interest to health professionals, healthcare resource providers, health policy planners, Health Departments and the Ministry of Health, healthcare companies, health sector industries, prosecutors, attorneys, judges, and thus everyone who is seriously involved with their own health, the health of their nearest and dearest, and the health of society as a whole.

It should also be pointed out that the activities of the Cochrane Collaboration must be independent of the interests of companies that aim to make profits coming from the field of health, so that it can retain its impartiality. For a long time, our colleagues and the entities that represent them have been calling for independent scientific activities without conflicts of interest. The Colloquium does not accept sponsorship from such companies, not because it does not consider them to have a very important role, but to be able to be in a position to critically evaluate their products. The search for efficiency and safety within the field of health is also of interest with regard to maintaining a healthy consumer market for all participants.

The Colloquium is being organized by the Brazilian Cochrane Center and by the Regional Library of Medicine (Biblioteca Regional de Medicina, BIREME). Make a date in your diary for the Cochrane Colloquium in São Paulo, from October 23 to 27, 2007 (www.centrocochranedobrasil.org.br, www.colloquiumbrasil.info).

